# Performance enhancement of high order Hahn polynomials using multithreading

**DOI:** 10.1371/journal.pone.0286878

**Published:** 2023-10-25

**Authors:** Basheera M. Mahmmod, Wameedh Nazar Flayyih, Zainab Hassan Fakhri, Sadiq H. Abdulhussain, Wasiq Khan, Abir Hussain

**Affiliations:** 1 Department of Computer Engineering, University of Baghdad, Baghdad, Iraq; 2 School of Computer Science and Mathematics, Liverpool John Moores University, Liverpool, United Kingdom; 3 Department of Electrical Engineering, University of Sharjah, Sharjah, United Arab Emirates; Vinnytsia National Technical University, UKRAINE

## Abstract

Orthogonal polynomials and their moments have significant role in image processing and computer vision field. One of the polynomials is discrete Hahn polynomials (DHaPs), which are used for compression, and feature extraction. However, when the moment order becomes high, they suffer from numerical instability. This paper proposes a fast approach for computing the high orders DHaPs. This work takes advantage of the multithread for the calculation of Hahn polynomials coefficients. To take advantage of the available processing capabilities, independent calculations are divided among threads. The research provides a distribution method to achieve a more balanced processing burden among the threads. The proposed methods are tested for various values of DHaPs parameters, sizes, and different values of threads. In comparison to the unthreaded situation, the results demonstrate an improvement in the processing time which increases as the polynomial size increases, reaching its maximum of 5.8 in the case of polynomial size and order of 8000 × 8000 (matrix size). Furthermore, the trend of continuously raising the number of threads to enhance performance is inconsistent and becomes invalid at some point when the performance improvement falls below the maximum. The number of threads that achieve the highest improvement differs according to the size, being in the range of 8 to 16 threads in 1000 × 1000 matrix size, whereas at 8000 × 8000 case it ranges from 32 to 160 threads.

## Introduction

Moment theory is a powerful tool in the areas of image processing, pattern recognition, and computer vision applications [1]. Signals are described using scalar values called moments (one, two, or more dimensions). A set of polynomial basis functions is utilized to compute moments. These basis functions are used to convert signals, voice or images, to the transform domain [2, 3]. To deal with the problem of pattern identification, Hu [4] introduced geometric moments and moments invariants. The proposed momemnts are not orthogonal, which result in numerical difficulties [5].

Continuous moments could be determined using continuous orthogonal polynomials like Zernike [6] or by using Tchebichef with altered radius [7]. Continuous moment functions can be incorrect due to two common types of errors: image coordinate transformation and continuous integral approximation. [8]. Due to the utilization of discretization and approximation throughout the process of image reconstruction, the obtained image will be imperfect [9].

In order to avoid the aforementioned constraints, Discrete Orthogonal Polynomials (DOPs) have been concentrated on by the researchers. This is due to their remarkable image reconstruction features [8–10]. In addition, 1D and 2D signals can be represented by discrete orthogonal moments (DOMs) without redundancy. Also, they have great energy compaction, and spectrum resolution characteristics [11–14]. Signal representation and feature extraction have recently been used to discrete Tchebichef polynomials [15, 16], discrete Hahn moments [17], and discrete Krawtchouk moments [18, 19]. It is note worthy that the DOPs are utilized for solving linear functional differential equations [20].

DOPs’ are solid owing to their significant features, which involve localization, energy compression, watermarking, signal extraction features, numerical stability, efficient data processing, and resilient data analysis [3, 21–25]. At the same time, the vital characteristics of majority of DOMs are not applied to large-sized images which is due to limitation in the computation of polynomials [26].

The DOPs limitations such as overflow, the instability of the polynomial values, and the high computational complexity have resulted in this constraints. Therefore, an improved recurrence technique for generating higher orders are being improved, for example Tchebichef [16] and Krawtchouk [18] polynomials. Recently, Researchers have considered other DOPS, for example Charlier polynomials [27] and Hahn polynomials [26].

The computation of DOP coefficients and the propagation of errors have been simplified by using the recursive algorithms [28, 29]. Regarding degree *n* either a single or double recursive formula can be employed. It also considers the time or spatial coordinate. To overcome the instability in the numerical values, the DOP coefficients should be calculated in the direction of the variable *n*. However, when the size of one or two dimensional signals turns large these calculations become inefficient. Since small values are assumed for the squared norm of the scaled Tchebichef polynomials, the coefficients of Tchebichef polynomials, for example, suffer from instabilities in the numerical values. To solve the aforementioned problem, the recurrence method in the *x*-direction was introduced by Mukundan [9]. After this study, this issue has received significant attention in many studies, for example, [28].

Generally, there has been a lot of focus on computation cost [30, 31]. It is considered as a key element which assist in ill-conditioning. For this reason, large number have considered it [32, 33]. This drawback is addressed in [34] by using a rapid and efficient computation method for Meixner moment coefficients. Another research introduced a fast and stable approach of Tchebichef moments for higher polynomial order, this is performed by combining the recurrence algorithms in the *n* and *x* directions [16]. Daoui et al. [26] utilized Gram-Schmidt orthogonalization procedure to reduce numerical error propagation. However, this technique is relatively slow.

This paper proposes a novel technique inspired by discrete orthogonal Hahn moments. Based on the literature, the three-term recurrence algorithms have been utilized in several existing works to tackle the problem of computational cost and propagation error due to gamma and binomial functions [35]. In [28], the n-direction recurrence algorithm was employed with an initial value starting at n, x = 0. The drawback of the algorithm presented in [28] (the recurrence algorithm in the n-direction) comes from starting the sets of initial values which are based on the initial value at *n*, *x* = 0. The initial sets are computable for a very restricted DHaP polynomials size and parameters. This results in a maximum computable polynomial size of 135. In other words, the limitation is due to the employed formula. In addition to the issue of high computational cost, algorithm used has numerical instability. To resolve the issue of the recurrence algorithm in the n-direction, the x-direction recurrence relation is adopted with a symmetry relation for equal values of the polynomials parameters [28]. Using the symmetry relation allows a reduction of 50% in the computed coefficients, which reduces the computation cost. Yet the recurrence relation in the *x*-directions has two limitations. The first limitation is that, according to the nature of the formula being utilized, the initial set becomes 0 when samples size or parameter values tends to be large. The second limitation is that when the degree of the polynomial increases, the coefficient values underflow because the initial value are less than 10^−324^, which equals zero for various values of the polynomials’ parameters. The highest possible order that can be calculated occurs at *n* = 1423. To overcome the limitation of previous recurrence algorithms, Daoui et al. [26] presented a technique based on the *n*-direction recurrence relation and the Gram-Schmidt orthonormalization process (GSOP). The utilization of the GSOP minimizes the numerical errors due to the use of the *n*-direction recurrence algorithm. However, the GSOP-based recurrence resolves the orthogonality of the DHaPs, but it has several limitations. First, the algorithm is unable to accurately calculate the coefficients of the DHaP when the parameters are not equal. Second, due to the technique used to calculate the initial values, the algorithm is still unable to generate DHaP for a wide range of DHaP parameters. Third, the nested loops of the GSOP algorithm result in a high computational cost, which in turn raises the number of processes required to compute the coefficients of the DHaP. Recently, a new mathematical model has been presented by [36], which can compute the DHaP’s initial value for a wide range of DHaP parameters values. In order to stabilize the computation of the DHaP coefficients, the algorithm also consists of two recurrence algorithms with adaptive thresholds. Although the algorithm in [36] can compute the coefficients of the polynomials more accurately than other algorithms, it still suffers from computation overhead.

In this paper, a fast approach for computing the DHaPs is proposed and applied to high orders. This work takes advantage of the multithread for the computation of Hahn polynomials coefficients. To take advantage of the available processing capabilities, independent calculations are divided among threads. The research provides a distribution method to achieve a more balanced processing burden among the threads.

This paper is organised as follows: in Section “Mathematical definition of DHaP and its moments” Preliminaries and current three-term recurrence algorithms are discussed. The proposed recurrence algorithm is presented in Section “Proposed Recurrence Algorithm”. In Section “Experimental Results”, the proposed recurrence method is evaluated by an experimental investigation. Finally, the paper is concluded in Section “Conclusion”.

## Mathematical definition of DHaP and its moments

This section presents the mathematical principles of the DHaP and their moments.

### The definition of DHaPs

The *n*th order of the DHaP is defined as [28]:

$$\begin{array}{c} H_{n}^{\alpha ,\beta }(x;N)=\frac{{\left(-1\right)}^{n}\;{\left(\beta +1\right)}_{n}\;{\left(N-n\right)}_{n}}{n!}\;{}_{3}F_{2}(\begin{array}{c} -n,-x,n+1+\alpha +\beta \\ \beta +1,1-N \end{array}|\;1), \end{array}$$ (1)

where _3_*F*_2_(⋅) is the generalised hypergeometric series denoted by:

$$\begin{array}{c} {}_{3}F_{2}(\begin{array}{c} a_{1},a_{2},a_{3}\\ b_{1},b_{2} \end{array}|\;c)=\sum_{k=0}^{\infty }\frac{{\left(a_{1}\right)}_{k}\;{\left(a_{2}\right)}_{k}\;{\left(a_{3}\right)}_{k}}{{\left(b_{1}\right)}_{k}\;{\left(b_{2}\right)}_{k}\;k!}{\left(c\right)}^{k} \end{array}$$ (2)

and (⋅)_*k*_ is the Pochhammer symbol [37].

The orthogonality of the DHaPs is satisfied as follows:

$$\begin{array}{c} \sum_{x=0}^{N-1}H_{n}^{\alpha ,\beta }(x;N)H_{m}^{\alpha ,\beta }(x;N)\omega \left(x\right)=\rho \left(n\right)\delta_{nm}\;, \end{array}$$ (3)

where *ρ* demotes the norm function of DHaP, *ω* represents the weight function of the DHaP, and *δ*_*nm*_ is the Kronecker delta. The norm and weight functions of the DHaP are defined as follows:

$$\omega (x)=\frac{\Gamma (\beta +x+1)\Gamma (N-x+\alpha )}{\Gamma (x+1)\Gamma (N-x)}$$ (4)

$$\rho (x)=\frac{{\left(\alpha +\beta +n+1\right)}_{N}\Gamma \left(\alpha +n+1\right)\Gamma \left(\beta +n+1\right)}{\left(2n+\alpha +\beta +1)\Gamma (N-n)\Gamma (n+1\right)}.$$ (5)

The *n*th degree of the weighted and normalized DHaP is given by

$$\begin{array}{c} {\hat{H}}_{n}^{\alpha ,\beta }(x;N)=H_{n}^{\alpha ,\beta }(x;N)\sqrt{\frac{\omega }{\rho }}. \end{array}$$ (6)

### The definition of DHaM

DHaMs are the projection of the signals on the basis of the DHaP. Suppose a 2D signal *f*(*x*, *y*) of size *N*_1_ × *N*_2_. Then, the DHaMs, Ψ_*nm*_, can be calculated:

$$\begin{array}{cc} & \Psi_{nm}=\sum_{x=0}^{N_{1}-1}\sum_{y=0}^{N_{2}-1}{\hat{H}}_{n}^{\alpha ,\beta }(x;N_{1}){\hat{H}}_{m}^{\alpha ,\beta }(y;N_{2})\;f\left(x,y\right) \end{array}$$ (7)

$$\begin{array}{c} n=0,1,\ldots ,N_{1}-1,\;\;\text{and}\\ m=0,1,\ldots ,N_{2}-1, \end{array}$$ (8)

To reconstruct back the 2D signal, image, to the spatial domain, the reconstructed signal $\hat{f}\left(x,y\right)$ can be computed as follows:

$$\begin{array}{c} \hat{f}\left(x,y\right)=\sum_{n=0}^{N_{1}-1}\sum_{m=0}^{N_{2}-1}{\hat{H}}_{n}^{\alpha ,\beta }(x;N_{1}){\hat{H}}_{n}^{\alpha ,\beta }(y;N_{2})\Psi_{nm}\\ x=0,1,\ldots ,N_{1}-1;\;\;\text{and}\;\;y=0,1,\ldots ,N_{2}-1. \end{array}$$ (9)

### Related work

It is well known that hypergeometric series defined in Eq (1) is computationally cost and shows imprecise precision of the polynomials coefficients; thus, the three term recurrence relations are used. The available recurrence relations with their analysis are discussed in this section.

#### The recurrence relation in the *n*-direction (RRnd)

The *n*th degree of the DHaP at the *x*th index is defined as follows [28]

$$\begin{array}{cc} & {\hat{H}}_{n}^{\alpha ,\beta }(x;N)=\frac{AB}{E}\;{\hat{H}}_{n-1}^{\alpha ,\beta }(x;N)+\frac{CD}{E}\;{\hat{H}}_{n-2}^{\alpha ,\beta }(x;N) \end{array}$$ (10)

$$\begin{array}{c} \;n=2,3,\ldots ,N-1,\;\;\text{and}\\ x=0,1,\ldots ,N-1, \end{array}$$ (11)

the recurrence relation parameters are defined by:

$$\begin{array}{c} A=x-\frac{2N+\gamma_{2}-2}{4}-\frac{\left(-\alpha^{2}+\beta^{2}\right)\left(2N+\gamma_{1}\right)}{4\left(2n+\gamma_{1}-2\right)\left(2n+\gamma_{1}\right)}\\ B=\sqrt{\frac{n\left(n+\gamma_{1}\right)\left(2n+\gamma_{1}+1\right)}{\left(N-n\right)\left(n+\alpha \right)\left(n+\beta \right)\left(2n+\gamma_{1}-1\right)\left(N+n+\gamma_{1}\right)}}\\ C=-\frac{\left(n+\alpha -1\right)\left(n+\beta -1\right)\left(N+n+\gamma_{1}-1\right)\left(N-n+1\right)}{\left(2n+\gamma_{1}-2\right)\left(2n+\gamma_{1}-1\right)}\\ D=\sqrt{\frac{n\left(n-1\right)\left(n+\gamma_{1}\right)\left(n+\gamma_{1}-1\right)\left(2n+\gamma_{1}+1\right)}{\left(\alpha +n)(\alpha +n-1)(\beta +n)(\beta +n-1)(N-n+1)(N-n\right)}}\times \\ \;\sqrt{\frac{1}{\left(\gamma_{1}+2n-3\right)\left(\gamma_{1}+N+n\right)\left(\gamma_{1}+N+n-1\right)}}\\ E=\frac{n(\gamma_{1}+n)}{\left(\gamma_{1}+2n-1\right)\left(\gamma_{1}+2n\right)}\\ \;\gamma_{1}=\alpha +\beta \\ \;\gamma_{2}=\alpha -\beta \end{array}$$ (12)

with initial values

$${\hat{H}}_{0}^{\alpha ,\beta }(x;N)=\sqrt{\frac{\omega (x)}{\rho (0)}}$$ (13)

$${\hat{H}}_{1}^{\alpha ,\beta }(x;N)=[-(\beta +1)(N-1)+x(\alpha +\beta +2)]\sqrt{\frac{\omega (x)}{\rho (1)}}.$$ (14)

The problem of the recurrence relation in the *n*-direction recurrence algorithm is due to the utilized initial values ${\hat{H}}_{0}^{\alpha ,\beta }(x;N)$ and ${\hat{H}}_{1}^{\alpha ,\beta }(x;N)$. These initial values bound the polynomial to low values of polynomial size *N*, where the largest size can be obtained is 135 samples which occurs at limited range of DHaP parameters, *α* = 20 and *β* = 20. This limitation occurs because of the utilized formulas. To resolve this problem, the complexity of the utilized formulas can be reduced; however, the recurrence relation in the *n*-direction still shows numerical propagation error [36].

#### The recurrence relation in the *x*-direction (RRxd)

To compute the DHaPs at the *x*th index with *n*th degree, the following recurrence is used [28]:

$$\begin{array}{cc} & {\hat{H}}_{n}^{\alpha ,\beta }(x;N)=\eta_{1}[\eta_{2}{\hat{H}}_{n}^{\alpha ,\beta }(x-1;N)+\eta_{3}{\hat{H}}_{n}^{\alpha ,\beta }(x-2;N)] \end{array}$$ (15)

$$\begin{array}{c} x=2,3,\ldots ,N-1,\;\;\text{and}\\ n=0,1,\ldots ,N-1, \end{array}$$ (16)

The recurrence relation coefficients *η*_1_, *η*_2_, and *η*_3_ are computed as follows:

$$\begin{array}{cc} \eta_{1}=\frac{\sqrt{\omega (x)}}{\tau (x-1)+\sigma (x-1)} & \sigma (x)=x(\alpha +N-x)\\ \eta_{2}=\frac{\tau (x-1)+2\sigma (x-1)-\lambda (n)}{\sqrt{\omega (x-1)}} & \tau (x)=(\beta +1)(N-1)-x(\alpha +\beta +2)\\ \eta_{3}=-\frac{\sigma (x-1)}{\sqrt{\omega (x-2)}} & \lambda (n)=n(n+\alpha +\beta +1) \end{array}$$ (17)

with initials

H^nα,β(0;N)=(1-N)n(n+βn)ω(0)ρ(n) (18)

$$\begin{array}{cc} {\hat{H}}_{n}^{\alpha ,\beta }(1;N) & =\frac{\left(n+\beta +1)(N-n-1)-n(N+\alpha -1\right)}{\left(\beta +1)(N-1\right)}\times \\ & \;\;\times \sqrt{\frac{\omega (1)}{\;\omega (0)}}{\hat{H}}_{n}^{\alpha ,\beta }(0;N). \end{array}$$ (19)

It is noteworthy that for the recurrence relation in the *x*-direction, symmetry relation [26] is employed to recude the computation cost:

$$\begin{array}{c} {\hat{H}}_{n}^{\alpha ,\beta }(x;N)={\left(-1\right)}^{n}{\hat{H}}_{n}^{\alpha ,\beta }(N-1-x;N)\;\text{for}\;\alpha =\beta . \end{array}$$ (20)

The utilization of the symmetry relation Eq (20) will reduce the computed coefficients to 50%. However, the recurrence relation in the *x*-direction still has two limitations. These limitations are:

1. The values of the ${\hat{H}}_{n}^{\alpha ,\beta }(0;N)$ tend to zero as the number of samples (*N*) increases and the values of the DHaP parameters becomes big. This is due to the formula used in Eq (18), and
2. The coefficient values of the DHaPs become underflowed as the polynomial degree (*n*) becomes large. This is due to the values of the initial becomes less than 10^−324^, which in turn goes to zero in different environments [36].

It is noteworthy that the DHaPCs become zero as the polynomial degree increases. For instance, the maximum non-zero coefficients occurred at *n* = 1423 when *N* = 1600 and *α* = *β* = 10 [36].

#### Recurrence relation based on Gram-Schmidt orthonormalization process (RRGSOP)

Daoui et al. [26] introduced their algorithm which is based on Gram-Schmidt orthonormalization process (GSOP), as well as n-direction recurrence relation to determine DHaP. In this case, GSOP is introduced to solve the issue instability in the DHaPCs. The proposed computes the initial sets ${\hat{H}}_{0}^{\alpha ,\beta }(x;N)$ and ${\hat{H}}_{1}^{\alpha ,\beta }(x;N)$. Recurrence relation in the *n*-direction is utilised to find the coefficients for *n* > 1 is then applied in order to reduce computational errors produced through the n-direction recurrence algorithm. Despite the fact that GSOP-based recurrence algorithm fulfil orthogonality condition, it has the following problems:

1. When *α* ≠ *β*, the algorithm fails to correctly identify the DHaP coefficients [26].
2. This algorithm is incapable to provide DHaP for a large range of values for *α* and *β*.
3. GSOP-based recurrence algorithm suffers from computational complexity as a result of the nested loops utilized to reduce the error for each polynomial degree. This can increase the number of operations required to calculate DHaP coefficients.

#### Hybrid recurrence algorithm

The authors in [36] provided a new algorithm for the purpose of solving the issues and limitations from previous algorithm. In this case the authors looked at two recurrence algorithms (which are the n- and x-recurrence relations) as well as adaptive threshold in order to be able to stabilize the generation of the DHaP coefficients. It should be noted that computational cost is considered important factor which is increased in this algorithm.

## Proposed recurrence algorithm

In this section, the proposed multi-thread recurrence algorithm for DHaPs is presented in details.

**Fig 1:**
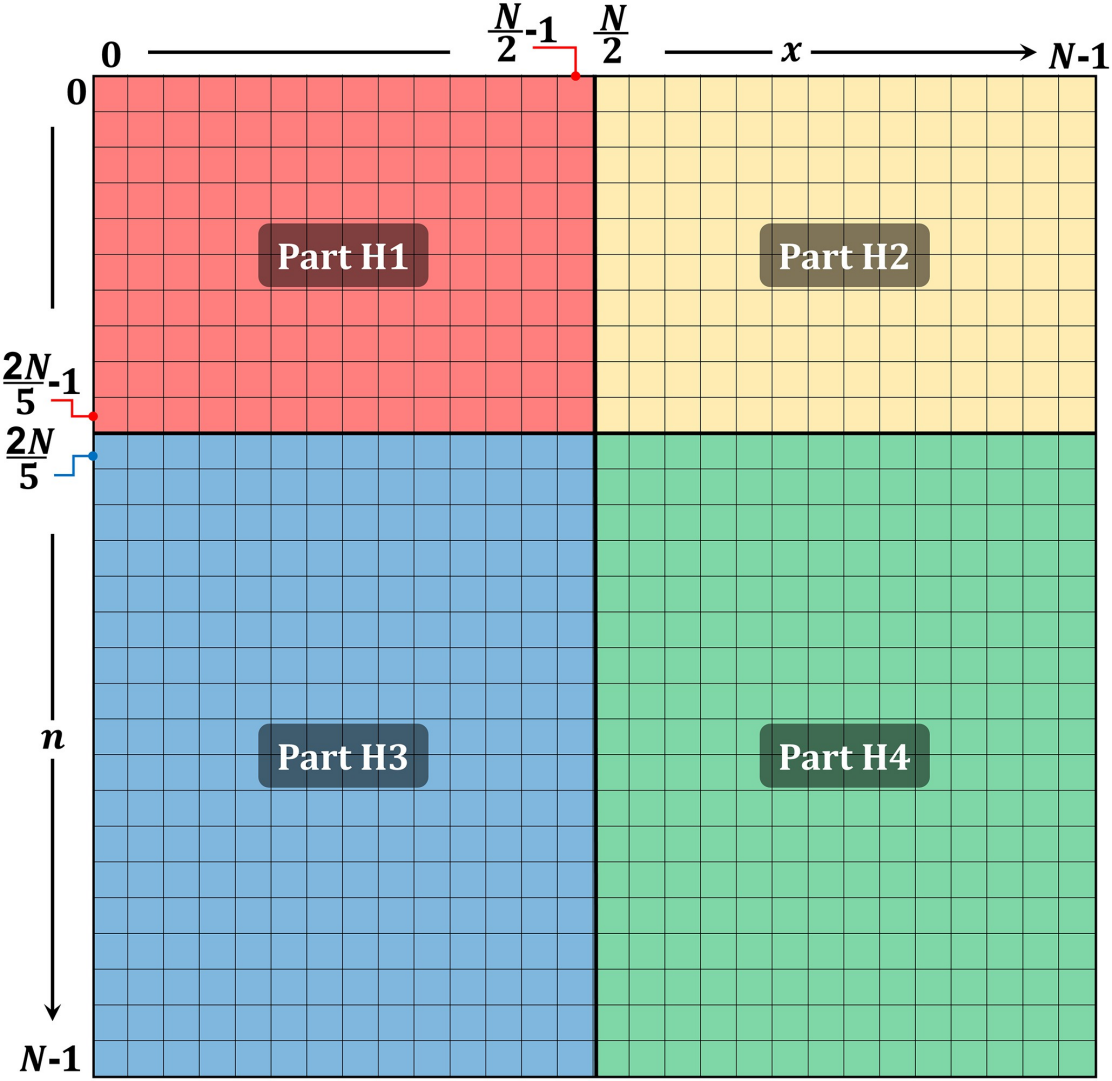
Partitions of the DHaPs.

**Fig 2:**
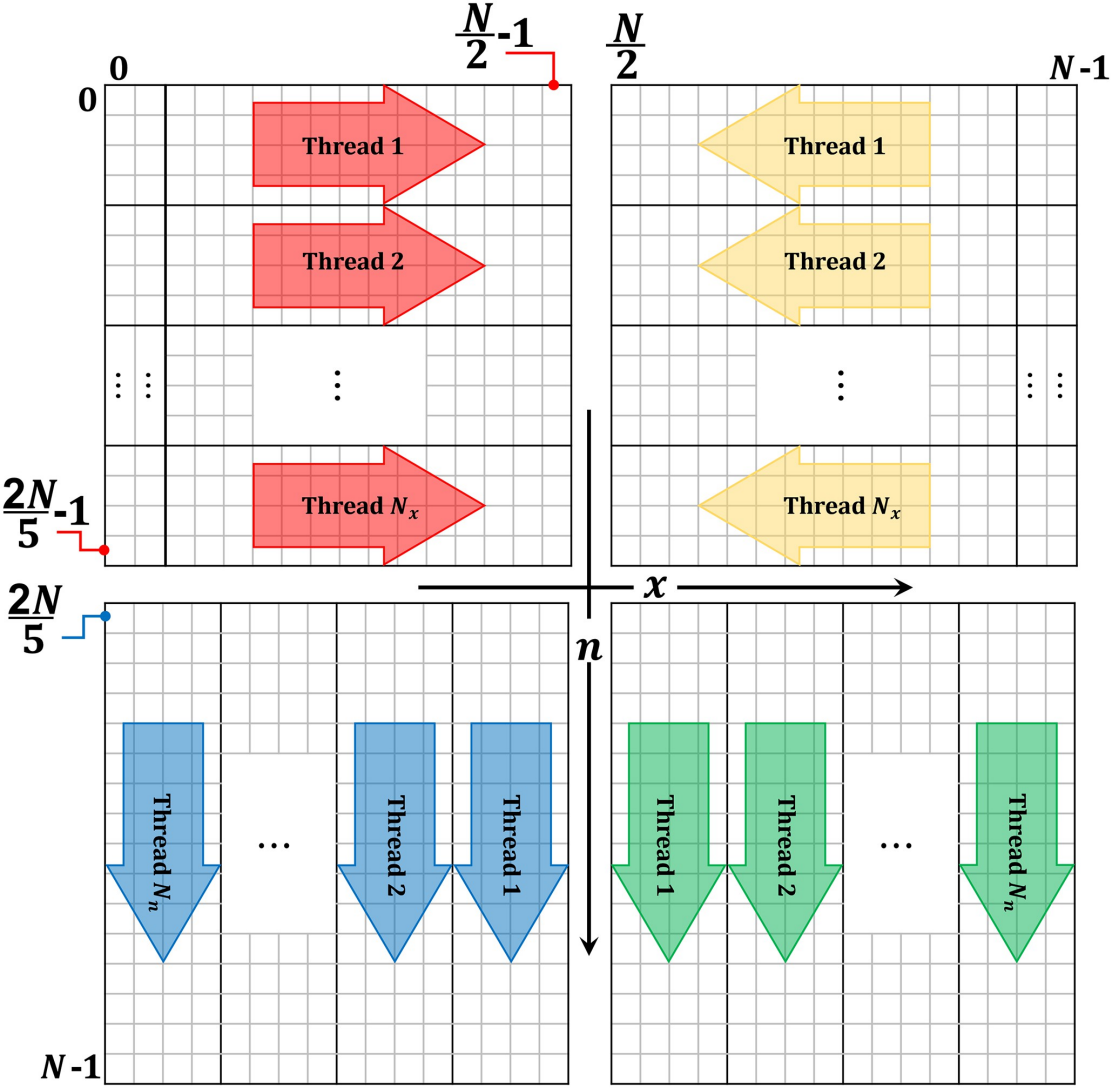
Steps of the proposed algorithm with threads.

The matrix of the DHaPs is divided into four parts (Part H1, Part H2, Part H3, and Part H4) similar to the partitions made in [36] (see Fig 1). First, computation of the first two columns at *x* = 0 and 1 are performed before the computation of the coefficients at part H1 (see Fig 2). Then, the proposed algorithm distributes the rows among a set of threads rather than the sequential processing of the H1 rows, which permits parallel processing of the coefficients. As every row is computed independently of the other rows. Part H2 is similarly divided among the same number of threads as part H1. Following the calculation of the last two columns, the threads start calculating the coefficient.

Furthermore, parts 3 and 4 (H3 and H4), begin respectively after parts H1 and H2 have been completed. The coefficients of the last two rows in part H1 are required to calculate part H3, however each column is independent of the others. Consequently, to perform parallel computation of the coefficients, the columns in part H3 are distributed among a set of threads. In the same way, after calculating part H2 coefficient, part H4 columns are processed using a set of threads.

For the case where *α* = *β*, there is a mirroring property that can be used to reduce the processing load. In the proposed algorithm, parts H2 and H4 coefficients are calculated inline with the part H1 and H3 calculations respectively. Using the available parallel resources, which are represented by multicores. The number of threads assigned to each scenario guarantees that parts H1 and H2 have the same processing burden. In parts H1 and H2 for 2*N*/5 rows, the number of rows for each thread will be bunch1 = 2*N*/5/*Th*; *Th* is the number of threads. The number of columns assigned for each thread in parts H3 and H4 will be *bunch*2 = (*N*/2)/*Th*.

## Experimental results

In order to evaluate the proposed threaded algorithm different matrix sizes are considered, namely 1000 × 1000, 2000 × 2000, 4000 × 4000, and 8000 × 8000. The unthreaded case in [36] is compared to some works in literature TRx [28], TRn [28], and TRGSOP [26] for the cases where *α* = *β* and *a* ≠ *b* to evaluate their performance. Tables 1 and 2 show the normalized performance of the different algorithms as compared to the unthreaded case [36]. For the case of *α* = *β*, the unthreaded case outperforms the other algorithms in all cases, except the TRn at 1000 matrix size. On the other hand, TRx outperforms the unthreaded case when *α* ≠ *β* for all matrix sizes, whereas TRn falls behind the unthreaded case at Large matrix sizes (4000 and 8000). TRGSOP show the worst performance at both *α* = *β* and *α* ≠ *β* in all matrix sizes. It should be noted that TRn, TRx, and TRGSOP are unstable in all the tested cases while the unthreaded algorithm is stable.

**Table 1:** Normalized performance of the different algorithms for *α* = *β*.

Size	TRn	TRx	TRGSOP	Unthreaded Case
1000	1.152	0.824	0.001540	1
2000	0.977	0.845	0.000444	1
4000	0.726	0.830	0.000281	1
8000	0.539	0.982	0.000159	1

**Table 2:** Normalized performance of the different algorithms for *α* ≠ *β*.

Size	TRn	TRx	TRGSOP	Unthreaded Case
1000	1.187	1.146	0.002090	1
2000	1.202	1.217	0.000570	1
4000	0.997	1.122	0.000360	1
8000	0.586	1.074	0.000170	1

The proposed threaded algorithm is evaluated and compared with the unthreaded case at the same cases of matrix sizes considered previously with different combinations of parameters *α* and *β*. In addition, different combinations of parameters *α* and *β* are considered as well. The proposed algorithm is based on the distribution of the computation load among independent threads to provide parallelism, thus enhancing performance. The effect of number of threads on the performance is evaluated by considering 2 threads up to 250 threads.

**Fig 3:**
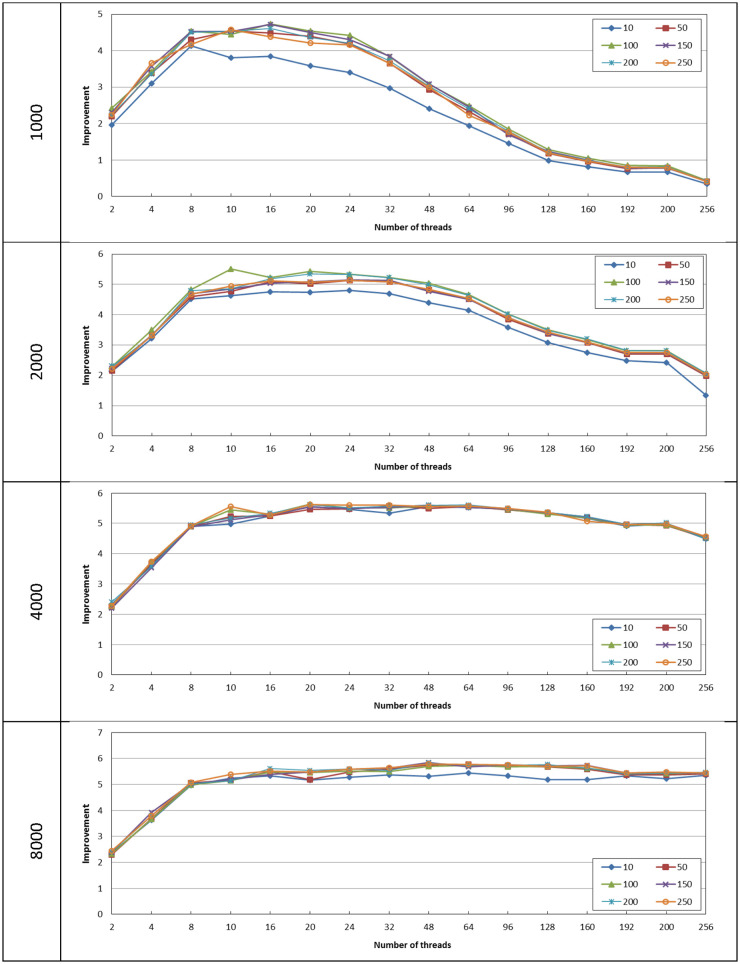
The improvement achieved by the proposed algorithm with respect to the unthreaded case for different size values with *α* = *β*.

Starting with the cases where *α* = *β* = 10, 50, 100, 150, 200, and 250, Fig 3 shows the performance improvement achieved by the proposed algorithm with respect to the unthreaded case. It can be noticed that the improvement has been slightly affected by the changes in the parameter values *α* = *β*, this is applicable at different number of threads and different matrix sizes. The highest difference can be observed at size = 1000 for *α* = *β* = 10 where it achieves up to 23% lower improvement as compared to the other parameter cases.

As can be noticed in Fig 3, all cases achieve about 100% improvement when the process is divided among two threads. This improvement linearly increases with the increase in the number of threads up to 8 threads. The threading technique has higher improvement as compared to the unthreaded case at higher matrix size, as it achieves up to 4.7, 5.5, 5.6, and 5.8 improvement at 1000, 2000, 4000, and 8000 matrix sizes, respectively. At the size of 1000, the improvement reaches its maximum value at number of threads ranging from 8 to 16. As the number of threads increases over the aforementioned range the improvement gradually declines and the performance worsens as compared to the unthreaded case when the number of threads exceeds 128. This can be linked to the exceeding overhead imposed by the high number of threads as compared to the useful processing carried out by each thread. This drop in improvement can also be noticed in the case of size = 2000, but at a slower slope and at worst case the improvement does not fall below 1.4. For the size of 4000 and 8000, as the number of threads is increased above 8 the improvement is maintained above 4.5 and 5 respectively.

**Fig 4:**
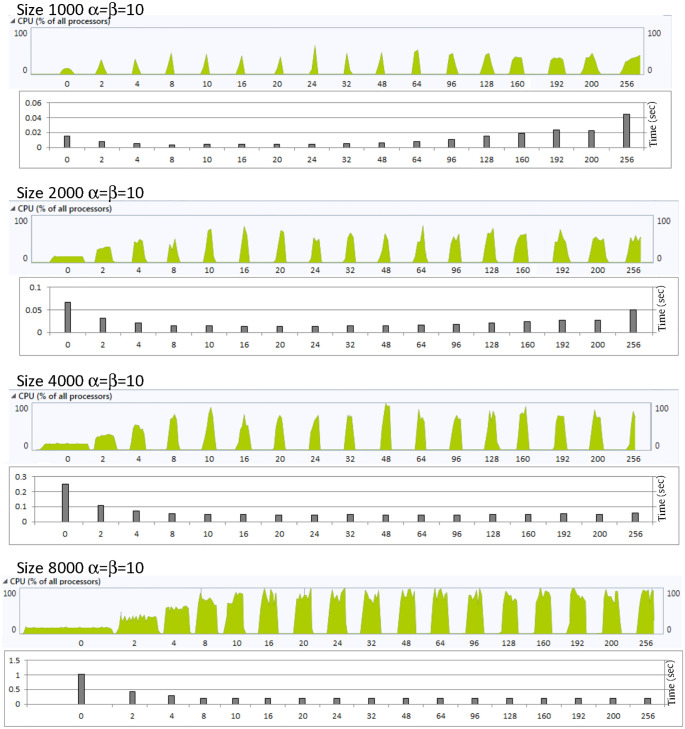
CPU utilization and the execution time for different values of polynomial size with *α* = *β*. Note that 0 in the x axis means unthreaded case.

Higher CPU utilization may imply an improved performance as more processing power is allocated to the algorithm which leads to reduced execution time. To some extent this relation is valid as can be seen in Fig 4. In the unthreaded case, the CPU utilization is limited to 13% which results in high delay. This CPU utilization increases as the number of threads increases and results in lower delays. This relation does not persist for matrix sizes 1000 and 2000, where the delay increases again when the number of threads exceed 16 and 32, respectively. This results in increased processing power with no performance gain which should be avoided.

**Fig 5:**
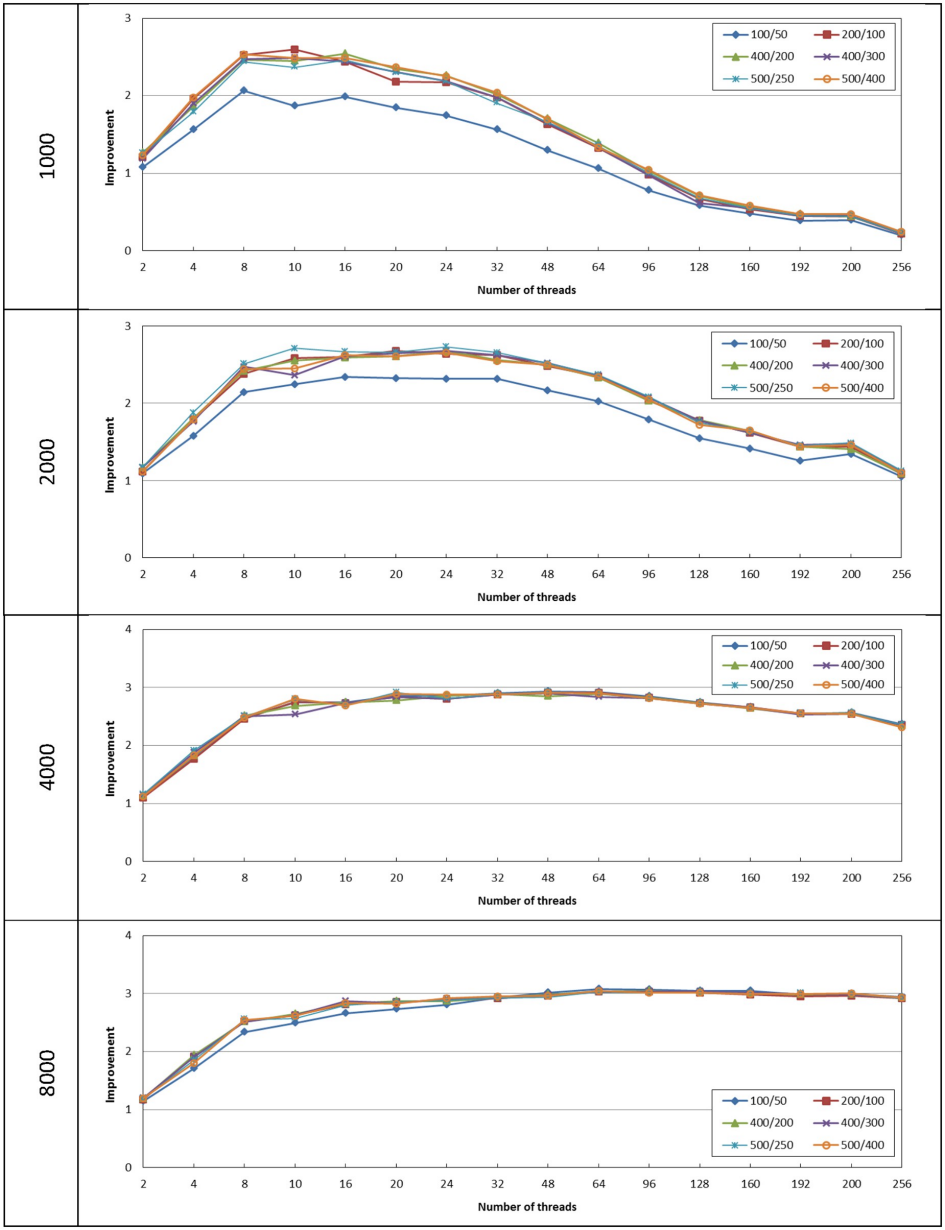
The improvement achieved by the proposed algorithm with respect to the unthreaded case for different size values with *α* ≠ *β*.

For the case where *α* ≠ *β*, the following values were considered (*α*/*β*) 100/50, 200/100, 400/200, 400/300, 500/250 and 500/400. Fig 5 shows the performance improvement achieved by the proposed algorithm with respect to the unthreaded case.

**Fig 6:**
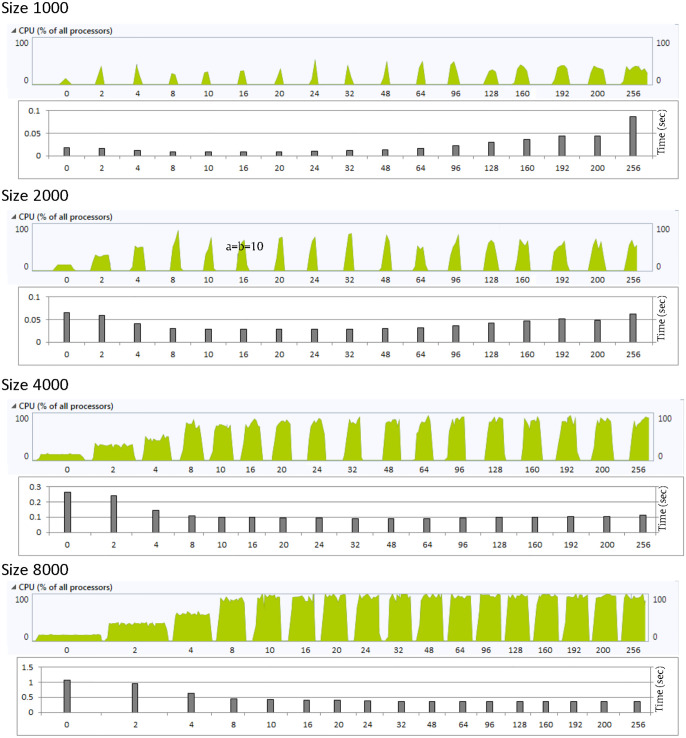
CPU utilization and the execution time for different values of polynomial size with *α* ≠ *β*. Note that 0 in the x axis means unthreaded case.

The threading technique achieves improvement with respect to the unthreaded case, and this improvement increases as the matrix size increases. It achieves up to 3.1 times improvement as compared to the unthreaded case at the size of 8000. At the size of 1000, the improvement reaches its maximum value at number of threads ranging from 8 to 16 which is similar to the cases where *α* = *β*. As the number of threads increases over the aforementioned range the improvement gradually declines and the performance worsens as compared to the unthreaded case when the number of threads exceeds 96. It reaches up to 5 times higher delay as compared to the unthreaded case, while the CPU usage is higher as shown in Fig 6. Any processing overhead with no performance gain should be avoided as it results in wasted power consumption.

It can be noticed that threading has higher improvement for the cases where *α* = *β* as compared to those cases where *α* ≠ *β*. In the former case, the coefficients in P2 and P4 are a mirror of those in P1 and P3 respectively. Thus, as soon as a coefficient in P1 and P3 is calculated, its mirror is immediately written and to avoid accessing the data again, mitigating memory reads penalties.

**Fig 7:**
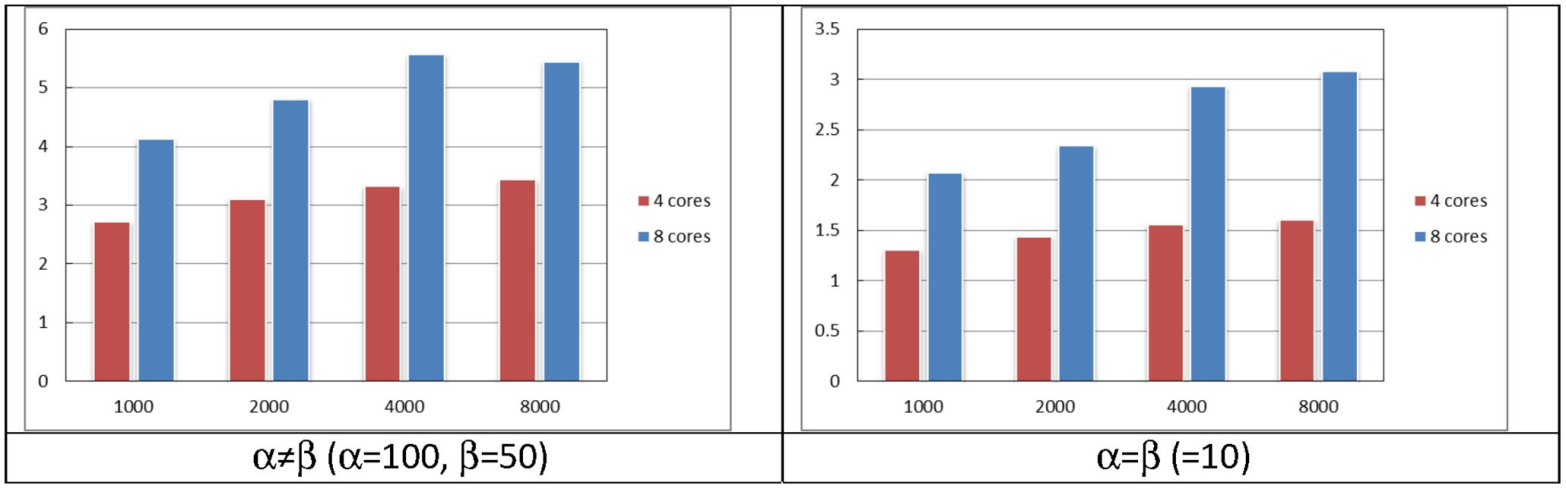
The effect of number of cores on the improvement for *α* = *β* and *α* ≠ *β*.

The number of cores affects the performance improvement of the threading technique. To evaluate this, the instead of enabling all of the eight logical cores as in the previous tests, only four logical cores were enabled. The tests were repeated with the new configuration and Fig 7 shows the effect of number of cores on the maximum improvement. The improvement in the four cores case is lower than that of the eight cores case by up to 48% when *α* ≠ *β* (*α* = 100 and *β* = 50). For the case where *α* = *β* = 10, the four cores show up to 40% lower performance improvement as compared to the eight cores. It should be noted that at the unthreaded case both, the four cores and eight cores, perform similarly. The unthreaded case uses one core only which represents 25% and 12.5% of CPU usage in the 4 cores and 8 cores, respectively.

## Conclusion

The paper proposed to exploit the parallelism in coefficient calculations allowing independent threads to process coefficients in parallel to enhance performance. The considered algorithm was analyzed at different matrix sizes, parameters’ values, and number of threads. The maximum performance improvement as compared to the unthreaded case was found at the case of *α* = *β* ranging from 4.7 at 1000 × 1000 matrix size to 5.8 at 8000 × 8000 matrix size. On the other hand, when *α* ≠ *β*, the maximum improvement was at the matrix size of 8000, achieving a maximum improvement of 3.1. The results show that the number of threads should be carefully selected to achieve the optimal improvement, since increasing this number over certain limits may lead to performance degradation due to the threading overheads. Small matrix size (1000 × 1000) reaches its maximum improvement when the number of threads ranges from 8 to 16 whereas the optimum range was from 32 to 160 at large matrix size (8000 × 8000). Reducing the number of cores from 8 to 4 reduces the performance by 48% and 40% for *α* = *β* and *α* ≠ *β*, respectively Other candidate algorithms exist that it is encouraged to be analyzed when the threading technique is introduced into them which requires identifying the independence within each algorithm in order to be parallelized.

## Abbreviations

The following abbreviations are used in this manuscript:

CPU: Central Processing Unit
DHaM: Discrete Hahn Moment
DHaPs: Discrete Hahn Polynomials
DOMs: Discrete Orthogonal Moments
DOPs: Discrete Orthogonal Polynomials
RRGSOP: Recurrence Relation Based on Gram-Schmidt orthonormalization process
RRnd: Recurrence Relation in the *n*-direction
RRxd: Recurrence Relation in the *x*-direction
